# Eco-chemical mechanisms govern phytoplankton emissions of dimethylsulfide in global surface waters

**DOI:** 10.1093/nsr/nwaa140

**Published:** 2020-06-23

**Authors:** Xuwei Deng, Jun Chen, Lars-Anders Hansson, Xia Zhao, Ping Xie

**Affiliations:** Donghu Experimental Station of Lake Ecosystems, State Key Laboratory of Freshwater Ecology and Biotechnology of China, Institute of Hydrobiology, Chinese Academy of Sciences, Wuhan 430072, China; Donghu Experimental Station of Lake Ecosystems, State Key Laboratory of Freshwater Ecology and Biotechnology of China, Institute of Hydrobiology, Chinese Academy of Sciences, Wuhan 430072, China; Department of Biology/Aquatic Ecology, Lund University, S-223 62 Lund, Sweden; State Key Laboratory of Vegetation and Environmental Change, Institute of Botany, Chinese Academy of Sciences, Beijing 100093, China; Donghu Experimental Station of Lake Ecosystems, State Key Laboratory of Freshwater Ecology and Biotechnology of China, Institute of Hydrobiology, Chinese Academy of Sciences, Wuhan 430072, China; Institute for Ecological Research and Pollution Control of Plateau Lakes, School of Ecology and Environmental Science, Yunnan University, Kunming 650091, China

**Keywords:** phytoplankton, lake ecosystem, biological regulation of global climate, dimethylsulfide, global surface oceans

## Abstract

The anti-greenhouse gas dimethylsulfide (DMS) is mainly emitted by algae and accounts for more than half of the total natural flux of gaseous sulfur to the atmosphere, strongly reducing the solar radiation and thereby the temperature on Earth. However, the relationship between phytoplankton biomass and DMS emissions is debated and inconclusive. Our study presents field observations from 100 freshwater lakes, in concert with data of global ocean DMS emissions, showing that DMS and algal biomass show a hump-shaped relationship, i.e. DMS emissions to the atmosphere increase up to a pH of about 8.1 but, at higher pH, DMS concentrations decline, likely mainly due to decomposition. Our findings from lake and ocean ecosystems worldwide were corroborated in experimental studies. This novel finding allows assessments of more accurate global patterns of DMS emissions and advances our knowledge on the negative feedback regulation of phytoplankton-driven DMS emissions on climate.

## INTRODUCTION

Dimethylsulfide (DMS) and its precursor, Dimethylsulphoniopropionate (DMSP), are majorly produced by algae [1–3] and also by a few species of intertidal plants [4], bacteria [5], cnidarians [6] and macro-invertebrates [7]. This biogenic-driven emission of DMS accounts for more than half of the total natural flux of sulfur to the atmosphere [8–10] and can be oxidized to large amounts of cloud-condensation nuclei, thereby causing reflection of sunlight (albedo), which in turn contributes to temperature reduction on Earth. This algal regulation of the climate is referred to as the ‘CLAW’ hypothesis [1]. Since this hypothesis was proposed, the role of DMS as an anti-greenhouse gas has become an important issue in the context of global warming [8,11,12]. Besides its anti-climate warming effects, DMS, or rather its precursor DMSP, serves as antioxidant and osmolyte in algal cells [3] and is also an important infochemical affecting trophic dynamics by attracting predators, such as seabirds, feeding on, for example, herbivorous crustaceans, thereby reducing the grazing pressure on phytoplankton [13]. Moreover, it is also an important source of carbon and sulfur to marine bacterioplankton [14,15] and may aid fish larva in locating their settlement habitat [16], thereby playing multiple fundamental roles in aquatic ecosystems.

In recent years, this hypothesis has been debated based on, for example, meteorological evidence [11,17]. In addition, the algal-driven emission of DMS has been estimated to range between 29 and 39 Tg S yr^−1^ [8–10], however, the relationship between DMS concentrations and algal biomass from field data has shown to be inconsistent or even controversial. For example, the relationship has been reported as unclear [1], contradictory [17], positive [18–20], negative [21,22], as well as absent [23] (Supplementary Fig. 1). Such uncertain relationships affect the calculation of DMS flux based on the algal biomass. In most models, the fluxes of DMS are obtained from small- or medium-scale field observations [24,25], jeopardizing our understanding of the mechanisms controlling DMS emissions, and is thus making it difficult to accurately predict future effects of  DMS on climate change and the global sulfur cycle [17].

The production and decomposition rates of DMS are critical to its concentrations in natural waters. DMS production from phytoplankton is affected by a variety of eco-physiological processes [8,26–28], such as ocean acidification [8,28,29], bacterial taxa and its decomposition pathway (lyase pathway or demethylation pathway of DMSP) [15,26,27], nitrogen-to-phosphorous ratio and nitrogen limitation [30,31], zooplankton grazing on phytoplankton [13] and also by the phytoplankton community composition [32]. It has been estimated that ∼90% of the DMS production is degraded through microbial decomposition [33,34]. Together with the degradation of photochemical oxidation [35,36], the DMS amount emitted from surface oceans to the atmosphere is <10% of the DMS production by phytoplankton in the ocean. Together, this indicates that the relationship between DMS concentration and phytoplankton is complex.

Therefore, in order to disentangle the contradiction between DMS concentrations and phytoplankton in previous studies, we have here conducted a field study using 100 shallow freshwater lakes (Supplementary Fig. 2) where we quantified DMS concentrations, phytoplankton biomass (Chlorophyll-a, Chl-a) and various environmental parameters, such as pH, transparency, dissolved oxygen, conductivity and so on. We also collected data of DMS and Chl-a simultaneously from a global sea-surface DMS database [37] (Supplementary Fig. 3), covering a wide range of phytoplankton biomasses in both oceans and freshwaters. We found a hump-shaped relationship between DMS and algal biomass due to intensive DMS degradation when the pH exceeded 8.1 (induced by active algal photosynthesis), which was furthermore validated by a series of laboratory experiments.

## RESULTS

### Global-scale relationships between DMS concentrations, phytoplankton and pH

**Figure 1.**
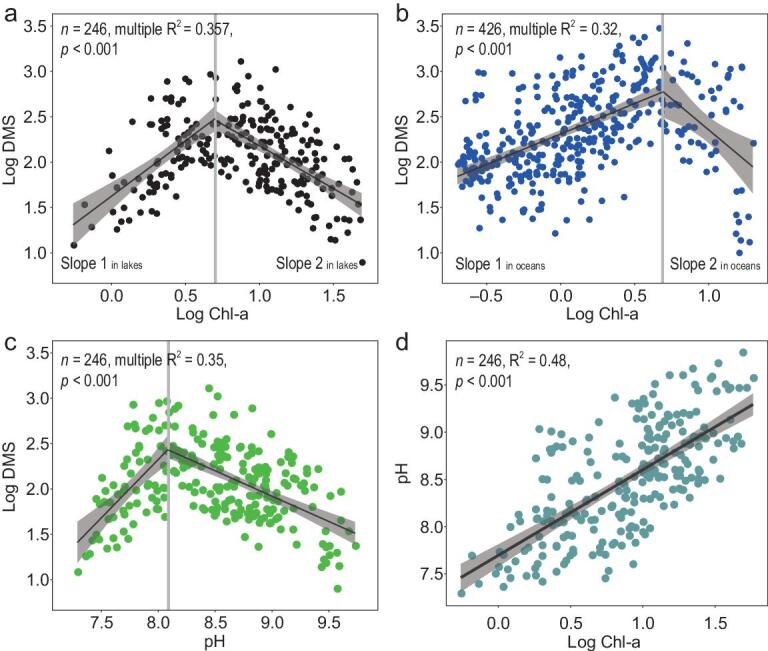
Quantitative relationships between DMS, Chl-a and pH in the 100 lakes in the eastern plain of China and the global surface oceans. (a) Relationships between LogChl-a and LogDMS in the 100 lakes, with a breakpoint of DMS at Chl-a = 5.04 ± 0.45 mg m^−3^, slope 1 in lakes = 1.22 and slope 2 in lakes = –1.00. (b) Relationships between LogChl-a and LogDMS in ocean surface water, with a breakpoint of DMS at Chl-a = 4.91 ± 0.66 mg m^−3^, slope 1 in ocean = 0.62 and slope 2 in ocean = –1.11. (c) Relationships between pH and LogDMS in the 100 lakes, with a breakpoint of DMS at pH = 8.09 ± 0.05. (d) Relationships between LogChl-a and pH in the 100 lakes.

We found a significant regression between log-transformed Chl-a and DMS concentrations in the 100 sampling lakes, with a breakpoint at Chl-a = 5.04 ± 0.45 mg m^−3^ (Fig. 1a). A similar segmented regression was also found in oceans, with a breakpoint at Chl-a = 4.91 ± 0.66 mg m^−3^ (Fig. 1b).

Based on previous studies [1,19,24,25], we expected a positive linear relationship between DMS and Chl-a [1,17,38], but this hypothesis was inconsistent with our field observations (Fig. 1a and b). Similarly, previous studies have shown that ocean-surface-water DMS concentrations are positively correlated with Chl-a at levels <3.5 mg m^−3^ [18,19], whereas others have reported a negative correlation at Chl-a levels >6.0 mg m^−3^ [21]. Hence, no previous study has covered a sufficient range of algal biomasses to draw an accurate conclusion [22,23]. Our study, allowing for novel conclusions, included Chl-a concentrations from 0.55 to 58.0 mg m^−3^ in lakes and from 0.055 to 39.08 mg m^−3^ in global surface oceans (Fig. 1a and b), representing a considerable range in Chl-a across surface waters worldwide [39]. Moreover, we can, for the first time, demonstrate the strikingly similar relationships between DMS and Chl-a in both fresh and ocean waters (Fig. 1a and b), suggesting a solid and global relationship between DMS and Chl-a in aquatic environments. However, the initial increasing slope of the regression between Chl-a and DMS in freshwaters was about twice as steep as in oceans (1.22 and 0.62, respectively; Fig. 1a and b), suggesting that DMS concentration increases faster in lakes than in oceans before reaching the pH breakpoint. This indicates that DMS production per unit Chl-a was higher in lakes than in oceans, which is seemingly contrary to the average DMS concentrations in the lakes and global oceans (175 and 379 ng L^−1^, calculated from Supplementary Tables 1 and 2, respectively). However, the intercept of DMS was higher in oceans than in lakes, indicating that there may be more sources of DMS at low Chl-a concentration in marine than in freshwater environments.

In our study lakes, a strong relationship was found also between DMS concentrations and pH, with a breakpoint at pH = 8.09 ± 0.05 (Fig. 1c). Similarly, there was a strong positive relationship between pH and Chl-a (Fig. 1d). Since the global sea-surface DMS database lacks pH data [37], we were unable to examine the relationship between pH, DMS and Chl-a in oceans. However, it is well known that, since algal photosynthesis increases pH, there is a positive relationship between pH and Chl-a also in oceans [40]. Hence, in accordance with our findings in freshwaters, some previous ocean studies report a positive correlation between pH and DMS, showing peak DMS concentrations at a pH of ∼8.1 [8,28,41] (Supplementary Fig. 4). This strongly suggests a similar relationship between DMS and pH in oceans as in freshwaters.

### DMS decomposition at increased pH

**Figure 2.**
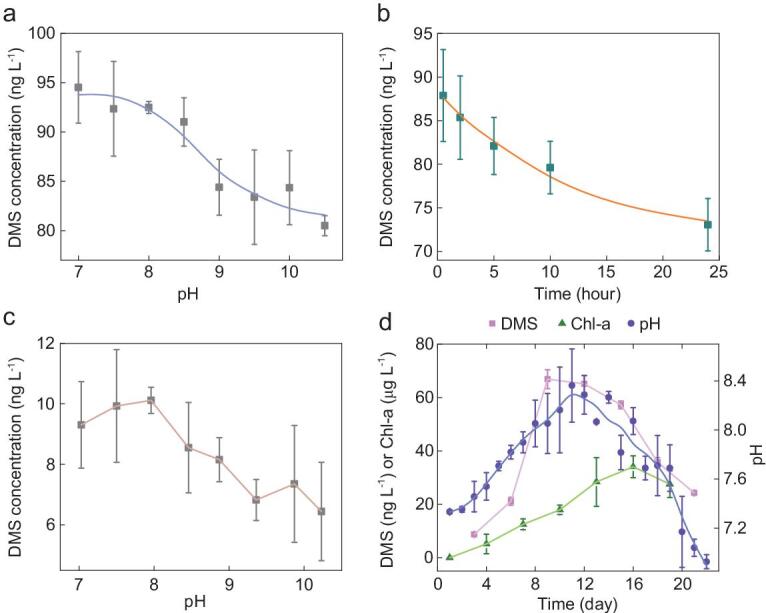
The changes in DMS concentrations in different alkaline solutions over time and in different algal cultures. (a) Changes in DMS with an initial concentration at 100 ng L^−1^ at eight different levels of pH solutions within 0.5 hours. (b) Continuous-integration changes in DMS with an initial concentration of 100 ng L^−1^ in 8 different pH solutions at 0.5, 2, 5, 10 and 24 hours. (c) The productions of DMS at eight different pH solutions (pH = 7, 7.5, 7.96, 8.46, 8.87, 9.36, 9.87 and 10.23) with similar algal biomass (*Phormidium foveolarum*, 1.032 × 10^6^ cells L^−1^) for 2 days. (d) The changes in DMS, Chl-a and pH in the verification test of *Emiliania huxleyi* over 22 days with axenic f/2 Medium solution.

Our observations from both lakes and oceans suggest that phytoplankton biomass and DMS emission initially increase with pH, but that DMS release shows a tipping point at a pH of ∼8.1. To experimentally test whether higher pH will lead to those general results from natural environments, pure DMS standard solution was diluted to a concentration of 100 ng L^−1^ with eight different pH levels ranging from 7 to 10.5 in the laboratory. Accordingly, we found that DMS concentrations decreased when pH increased to >8.0 (Fig. 2a) and that the decay rate of DMS was rapid during the first 5 hours in alkaline solutions and then slowed down, with a reduction of ∼30% after 24 hours (Fig. 2b). Hence, the mechanistic laboratory test corroborates the field data by showing that DMS is decomposed or converted into other compounds in alkaline waters, with a threshold pH at ∼8.0, where DMS decomposition accelerates considerably.

### DMS production by algae at different pH

We performed further verification tests in order to explore the relationships between algae, DMS production and pH using cultured algae. In the first experiment, an axenic freshwater alga, *Phormidium foveolarum*, was cultured with controlled biomass at different pH levels. DMS concentrations increased from 9.31 to 10.12 ng L^−1^ in the algal culture when pH increased from 7.0 to 8.0, but then decreased to 6.44 ng L^−1^ as pH increased further from 8.0 to 10.25 (a 36.4% decrease) (Fig. 2c). Hence, this assessment of the relationship between DMS and pH was also consistent with our field observations (Fig. 1a and b).

Moreover, in an axenic culture of the marine alga *Emiliania huxleyi*, which was cultured for 22 days, we found similar trends of DMS, Chl-a and pH with a breakpoint in DMS emission at pH = 8.06 (Fig. 2d), further strengthening the global and continental relationships identified in lakes and oceans (Fig. 1a and b).

### Global patterns in annual mean DMS concentration in oceans

**Figure 3.**
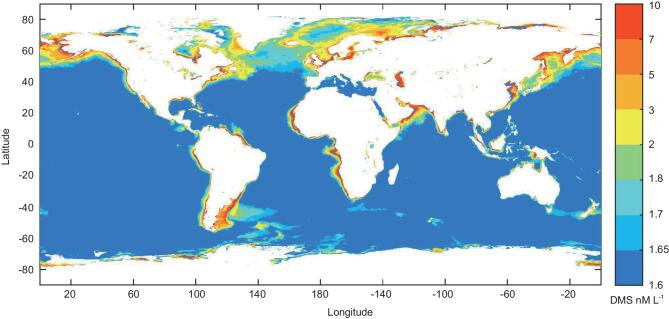
Global pattern of annual mean DMS concentrations (nM L^−1^) in surface oceans based on the relationship between DMS and Chl-a in Supplementary Fig. 5 and the average MODIS Chl-a concentration from 2003 to 2016. The DMS concentrations were high in the coastal regions (7–10 nM L^−1^) but low in open oceans (1.6–1.7 nM L^−1^).

Based on the relationship between DMS and Chl-a (Supplementary Fig. 5), we depicted the spatial pattern of DMS concentration in the global oceans by using 14-year (2003–16) averaged monthly composites of MODIS Chl-a data [42]. The results showed high-level DMS concentration (7–10 nM L^−1^) in coastal regions and it decreased to 1.6–1.7 nM L^−1^ in the open oceans (Fig. 3). Accordingly, an average sea-surface DMS concentration was estimated to be 1.92 nM L^−1^, falling into the range of previous studies [19,43–46] (Supplementary Table 3). Generally, DMS concentrations were higher in the coastal regions than in the open oceans. Concentrations were also high at northern high latitudes, with a decreasing trend towards the equatorial and subequatorial oceans.

## DISCUSSION

In the present study, the global pattern shows an increase in DMS concentration as phytoplankton production increases up to a pH of ∼8.1, but a decline when algal photosynthesis increases further. Undoubtedly, pH contributes greatly to the decline in DMS concentration as pH exceeds 8.1, although other environmental factors may also be involved in the decomposition of DMS. For example, as a reductive compound, DMS can be oxidized by microbes [33,34], metal ions, UV irradiation and photosensitizers [36]. Moreover, it was unclear in previous studies whether a high pH decreased DMS yields of the producers or not. In the present study, we observed DMS reductions of 30% and 36.4%, respectively, in pH-controlled solutions (Fig. 2b) and in pH-controlled freshwater algal cultures(Fig. 2c), suggesting that the reduction in DMS at a pH >8.1 was mainly caused by pH decomposition, but also due partly to lower DMS production by producers at the higher pH. Thus, our study first describes a hump-shaped relationship between DMS and phytoplankton in freshwater lakes, and then identifies a quite similar pattern in oceans, and the relationship is mainly formed by algal photosynthesis-driven DMS degradation at pH value >8.1.

Based on the hump-shaped relationship (Supplementary Fig. 5) and remote sensing data [42], the spatial pattern of global DMS was roughly similar to previous results, based either on spatial interpolation of DMS-concentration data (*n* = 47 000) in the global sea-surface DMS database [37] or on empirical relationships with Chl-a concentrations [43]. However, we corrected DMS concentrations at high algal biomass (Supplementary Fig. 5) and our results (estimated to be 1.92 nM L^−1^) rank in the middle of previous estimates (1.5–2.5 nM L^−1^) (Supplementary Table 3). Thus, the global pattern of DMS concentrations provided here is likely more precise than in previous studies [19,43–46].

In a broader context, our results suggest that, at higher pH, DMS is broken down, leading to a reduced DMS concentration in natural waters. In high productive waters, e.g. mainly in freshwaters and coastal areas of the oceans, high photosynthesis rates may elevate the pH far above 8.1, thus leading to a decline in DMS through pH-driven degradation (Fig. 1). Similarly, our results show that the DMS concentrations would also decrease with a decline in pH from 8.1 to 7 (Figs 1 and 2). Our results (Figs 1 and 2), together with the facts that oceans are facing anthropogenically induced acidification and that the acidity in polar oceans is changing at more than twice the global average [8,47], suggest that average DMS concentrations in the global oceans, particularly at the poles, will likely decline. Hence, further studies are needed to assess the DMS emissions from global surface oceans in the context of ocean acidification.

In addition to enhanced degradation of DMS at high pH, pH changes may also affect phytoplankton that produce DMSP, thereby altering DMS production. A few experimental studies addressing this issue have been performed, although the results are not consistent. For example, when pH declined from 8.3 to 7.49, the algal DMS production declined by ∼50%–60% [28,29,41], while the DMSP production from algae was found to be unaffected by pH [28], elevated at low pH [41] or decreased at low pH [29]. On the other hand, the effect of high pH (>8.1) on DMS/P production is unknown. It is also important to know how pH changes may affect microbial-mediated DMSP decomposition. Bacterial abundance and productivity have been found to increase at low pH, suggesting an increase in DMS and DMSP consumption [29,41], but pH-independent cases have also been reported [28]. DMSP-lyase activity showed great interspecific variations at the pH optimum, from pH 5 in a number of haptophyte *Phaeocystis* spp. and coccolithophore *Gephyrocapsa oceanica*, to pH 8 in the bacterium *Ruegeria lacuscaerulensis* and *Pseudomonas doudoroffii* and up to pH 10.5 in a *Phaeocystis* strain [29], which suggests that the relationship between pH and algal DMSP production or bacterial DMSP decomposition is rather complex and more comprehensive studies are needed in the future.

Another widespread role of DMS is its utilization by top predators in biological food webs, specifically among ocean-feeding seabirds when locating crustacean prey, which concentrate at sites with high algal abundances [13,48]. Hence, it is expected that, at sites where high amounts of DMS are released by phytoplankton, a high pH will lead to less exact location of crustacean prey, and thereby to less food, and likely, reproduction, by ocean birds—a notion open for further investigation. Hence, the close connection between pH and DMS demonstrated in our study may have a fundamental impact on several global-scale processes. These processes may act in concert or antagonistically and may even lead to synergistic effects as shown regarding other climate processes [49]. From this perspective, our novel data disentangle the mechanisms behind degradation of a major component of the global sulfur cycle and thereby open up for future studies on several crucial components of global-scale environmental changes.

**Figure 4.**
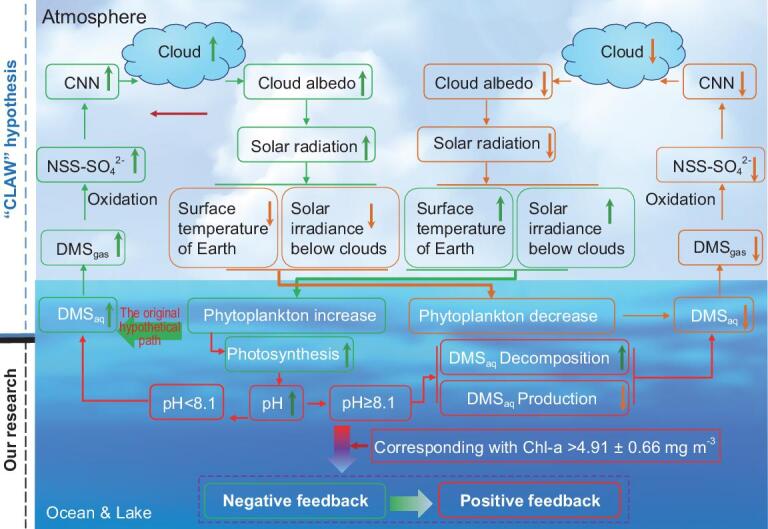
Schematic representation of how our study modifies and enriches the classic ‘CLAW’ hypothesis by finding the pH-dependent eco-chemical mechanisms governing phytoplankton emissions of DMS in global surface waters.

In brief, the ‘CLAW’ hypothesis proposed an ideal scenario of how biological processes regulate global climate through affecting cloud properties via sulfur-containing aerosols [1], yet most steps in the feedback loop of this hypothesis have not been validated [17], especially the relationship between DMS emission and phytoplankton biomass, which has, for a long time, remained a controversy. Our study solved this puzzle by finding a hump-shaped relationship between DMS concentration and phytoplankton biomass in both lakes and oceans, and further revealing the pH-dependent eco-chemical mechanisms governing phytoplankton emissions of DMS. Hence, our results proved that the ‘CLAW’ hypothesis is not perfect and were also a supplement to the hypothesis (Fig. 4). Overall, our results substantially enhance our understanding of the biological regulation of global warming by providing a more complete sulfur cycle and better sulfide-related global-climate-change models. Our results will also shed more light on the research of the ecological functions of DMS/P.

## METHODS

### Collection of field samples

We sampled 246 sites from 100 shallow freshwater lakes in the eastern plain of China during the summers of 2008 and 2009. Surface areas of the lakes ranged from 10.2 to 2933 km^2^, with a total area of ∼18 800  km^2^ and a longitude and latitude range of ∼10° × 10° (Supplementary Fig. 2). Water temperature, depth, pH, transparency, dissolved oxygen, conductivity, salinity, turbidity, oxidation-reduction potential and suspended solids were measured *in situ* by a multi-parameter water-quality sond (YSI 6600 V2, YSI Inc., Yellow Springs, USA) and Secchi disk. Physiochemical variables in the water samples, such as total nitrogen, nitrate, nitrite, ammonia, total phosphorus, phosphate, permanganate and Chl-a concentrations, were measured in the laboratory using chemical methods. The water samples for DMS detection were gravity-filtered using a replaceable film needle filter (by Glass microfiber filters, GF/C, 1.2 μm pore size, 47 mm diameter, Whatman, GE Healthcare Life Science, UK) under the same conditions in the field (a recovery test of DMS through gravity filtration is shown in the Supplementary Note), immediately, and 50 mL of filtered water of each sample were instantly frozen without bubbles and transferred to the laboratory, and then thawed at 4°C before DMS detection. We also collected 426 data sets of surface oceans, including simultaneously detected DMS and Chl-a, from the global database of sea-surface DMS from 20 contributors during 1981 and 2012 (Supplementary Fig. 3 and Supplementary Table 2). The relationships between environmental parameters and DMS concentration in lakes were analysed (Supplementary Fig. 6).

### Quantification of DMS

All DMS samples were detected by Eclipse 4660 Purge and Trap Sample Concentrator (OI Analytical Company, USA) and Gas Chromatography Mass Spectrometry (QP2010Plus, Shimadzu Corporation, Japan) with a Tenax trap (OI Analytical Company, USA) and an HP-5MS UI column (30 m × 0.25 mm × 0.25 μm, J&W Scientific, USA). All parameters and run settings were identical to our previous study [50].

### DMS-degradation experiments

Pure DMS standard (Tokyo Chemical Industry, purity: 99.0%) was diluted with methanol (Merck, HPLC grade) as the first DMS stock solution and then diluted with ultrapure water as the second DMS stock solution. To eliminate the possible interferences of biological or chemical factors (such as microorganisms and metal ions) on DMS degradation, tris-HCl (pH 7.1–8.9), glycine-NaOH (pH 8.6–10.6) and ultrapure water were used to modulate pH solutions. Then the second DMS stock solution was diluted to the above buffer solutions (pH 7.0, 7.5, 8.0, 8.5, 9.0, 9.5, 10.0 and 10.5, with three replicates) with a concentration of 100 ng L^−1^. Each solution was prepared before use and the DMS concentrations in the different pH solutions were detected at 0.5, 2.0, 5.0, 10.0 and 24.0 hours after being prepared.

### Algal culture experiments

Pure *P.foveolarum* (a DMS-associated algae in Cyanophyta, widely distributed in natural waters with a hypersaline and alkaline tolerance range) was obtained from the Freshwater Algae Culture Collection at the Institute of Hydrobiology (a verification experiment of DMS production from *P.foveolarum* is shown in the Supplementary Note) and cultured with axenic Blue-Green Medium 11 solution (BG11, according to the manufacturer’s instructions). At the beginning, we adjusted the BG11 culture medium to 8-pH levels (pH 7.0–10.25) with tris-HCl and glycine-NaOH, respectively, and then the algae were inoculated in the above mediums. Each treatment had 300 mL algae with ∼1.0 × 10^6^ cells mL^−1^ and each pH group had three replicates. All groups were cultured in an algal incubator (25°C, 12 hours light and 12 hours dark) for 2 days, and then the DMS and pH in each group were detected. Because of buffers and short duration, the pH of each culture medium changed little at the end.

Pure *E.huxleyi* (Prymnesiophyceae, Chromophyta) was obtained from the Center for Collections of Marine Algae at Xiamen University and three replicates were cultured with axenic f/2 Medium (without pH modulation according to the f/2 medium formula and with an initial pH of ∼7) solution for 22 days. DMS and Chl-a were detected every 3 days and pH was detected every day.

### Estimation of global sea-surface annual mean DMS concentration using MODIS data

We first established an empirical relationship between DMS and Chl-a concentrations in ocean surface water based on measured data at 426 sites from the global sea-surface DMS database [37]. The log-transformed DMS concentration showed a significant non-linear relationship with log-transformed Chl-a concentration (Supplementary Fig. 5, Adjusted *R*^2^ = 0.34, RMSE = 2.55 ng L^−1^) in the form of equation (1),

(1) \begin{equation*}{\rm{y\ }} = {y_0}\ + a{e^{\left[ { - 0.5\left( {\frac{{x - {x_0}}}{b}} \right)} \right]}}\end{equation*}

where *y*_0_ = 2.00, *a* = 0.83, *b* = 0.35 and *x*_0_ = 0.60. Parameter *y*_0_ represents a baseline (log-transformed) concentration throughout large areas of low-DMS waters. According to Anderson *et al.*, the model is unable to resolve DMS variability at low log_10_(Chl-a) [43] and thus we estimated global sea-surface DMS concentrations based on annual mean Chl-a concentrations, which were calculated based on 14-year (2003–16) monthly composites of MODIS Chl-a data with a regridded resolution of a 1/12° grid [42].

### Statistical analysis of data

We used piecewise linear regression by R version 3.2.5 (Comprehensive R Archive Network, TUNA Team, Tsinghua University) to confirm the relationship between DMS, Chl-a and pH from field data. SPSS version 20.0 (IBM corporation, Armonk, NY, USA), OriginPro 2015 (OriginLab Corporation, Northampton, MA 01060, USA), Statisticia version 6.0 (StatSoft Inc., Tulsa, OK 74104, USA) and ArcGis 10.0 (Esri China Information Technology Co. Ltd, Beijing, China) were used to analysis the data and draw the figures.

## Supplementary Material

nwaa140_Supplement_File
