# Supplementary material for: Eco-chemical mechanisms govern phytoplankton emissions of dimethylsulfide in global surface waters
Source: Natl Sci Rev. 2020 Jun 23;8(2):nwaa140. doi: 10.1093/nsr/nwaa140 (PMC8288430; doi:10.1093/nsr/nwaa140)
Supplement: nwaa140_Supplement_File [file nwaa140_supplement_file.docx]

**Supplementary Data for**

**Eco-chemical mechanisms govern phytoplankton emissions of dimethylsulfide in global surface waters**

Xuwei Deng^1^, Jun Chen^1^, Lars-Anders Hansson^2^, Xia Zhao^3^, Ping Xie^1,4*^

^1^Donghu Experimental Station of Lake Ecosystems, State Key Laboratory of Freshwater Ecology and Biotechnology of China, Institute of Hydrobiology, Chinese Academy of Sciences, Wuhan 430072, P. R. China.

^2^Department of Biology/Aquatic Ecology, Ecology Building, Lund University, S-223 62 Lund, Sweden.

^3^State Key Laboratory of Vegetation and Environmental Change, Institute of Botany, Chinese Academy of Sciences, Beijing 100093, P. R. China.

^4^Institute for Ecological Research and Pollution Control of Plateau Lakes, School of Ecology and Environmental Science, Yunnan University, Kunming 650091, P. R. China.

^*^Corresponding author. E-mail: [xieping@ihb.ac.cn](mailto:xieping@ihb.ac.cn)

This file includes:

Supplementary Note

Supplementary Figures 1-8

Supplementary Tables 1-6

Supplementary References

**Supplementary Note**

**A recovery test of DMS through the gravity filtration.** A bottle of water (turbidity 9.8 NTU) was sampled from Lake Donghu (E 114.3599, N 30.5462), and a DMS stock solution was spiked into the lake water, immediately, with a concentration of ca. 100 ng L^-1^. Then, part of the spiked lake water was loaded to three sample bottles (named NF-L1, NF-L2, NF-L3), and other spiked lake water was gravity filtered with a replaceable film needle filter by GF/C Glass microfiber filters three times, and the three filtrate were named F-L1, F-L2 and F-L3. After these preparations, the filtered and unfiltered samples were alternately detected in the purge and trap coupled to gas chromatography with mass spectrometry (P&T-GCMS). The results show that the recovery was about 100.7%, and the filtration loss rate is about -0.7%, and the relative standard deviation (RSD) of all the six samples (filtered and unfiltered samples together) was 1.9% (Supplementary Fig. 7 and Supplementary Table 4). This DMS recovery test shows that there was negligible difference in DMS concentrations between the two treatments, indicating that the gravity filtration method with a replaceable film needle filter by GF/C is feasible.

**A verification experiment of DMS production from *Phormidium foveolarum*.** A pure *Phormidium foveolarum* was obtained from the Freshwater Algae Culture Collection at the Institute of Hydrobiology. DMS, total DMSP, Chl-a and algal density in the medium (BG11) were detected at the beginning of the experiment (Supplementary Table 5 and Supplementary Fig. 8). And then, 2 mL medium were filtered with the above method (3 replicates), the filter and filtrate (2 mL) were filled into brown glass bottles (dealt with H_2_O_2_ and H_2_SO_4_ before using) with caps and PTFE-silicone septa, respectively. 2 mL 10 M NaOH and 40 mL ultrapure water were filled into the above bottles. We then screwed up the cap without bubbles in the bottles and shook them well. After standing overnight (12 h), the samples were anlayzed. The results showed that DMSP in both mediums and algal cells was confirmed (Supplementary Table 6) and thereby the used *Phormidium foveolarum* was confirmed to be a DMS producer.

**Supplementary Figures**


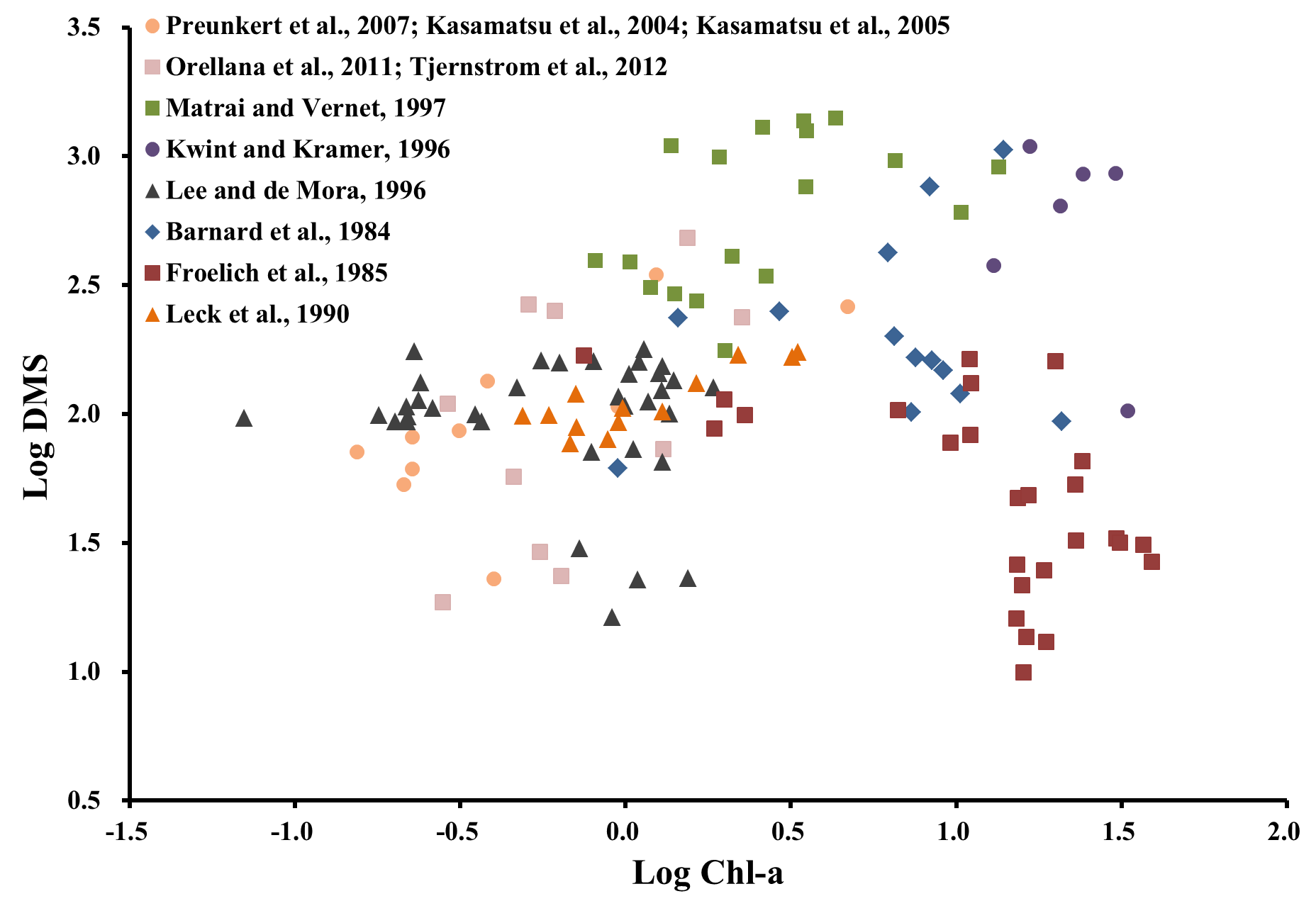


**Supplementary Figure 1. The relationships between DMS and algal biomass from previous studies: Orellana et al., 2011, Leck et al., 1990, Preunkert et al., 2007, Kasamatsu et al., 2004, Kasamatsu et al., 2005, Tjernstrom et al., 2012 showed positive [20, 51-55], Froelich et al., 1985 showed negative [21], Barnard et al., 1984, Matrai and Vernet, 1997, Kwint and Kramer, 1996, Lee and de Mora, 1996 showed absent [23, 56-58].**


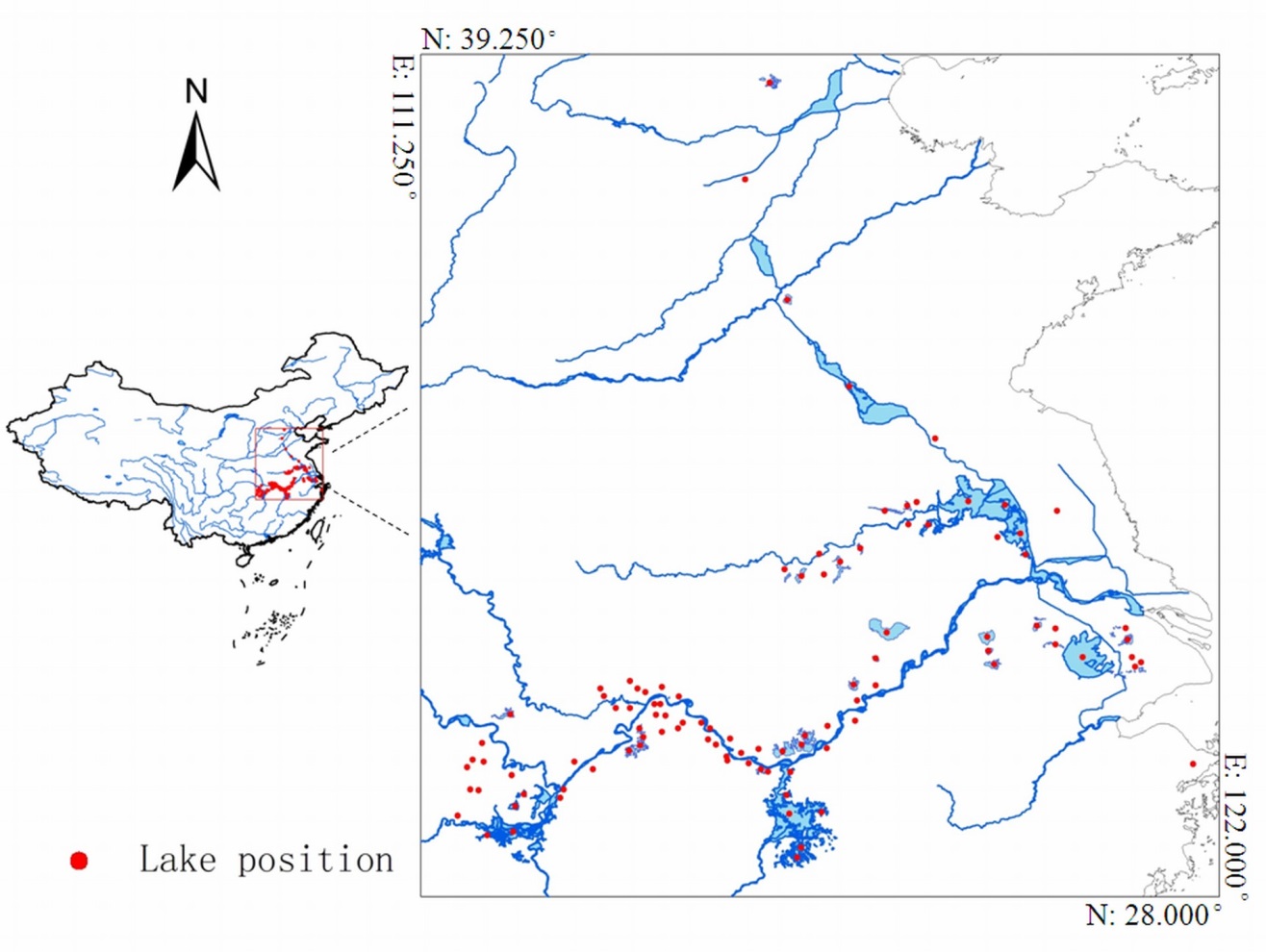


**Supplementary Figure 2. The locations of 100 shallow freshwater lakes in the eastern plain of China (246 sites), ranging in longitude from 111 ºE to 122 ºE, and in latitude from 28 ºN to 39 ºN. Surface areas of the lakes range from 10.2 to 2,933 km^2^, with a total area of about 18,800 km^2^.**


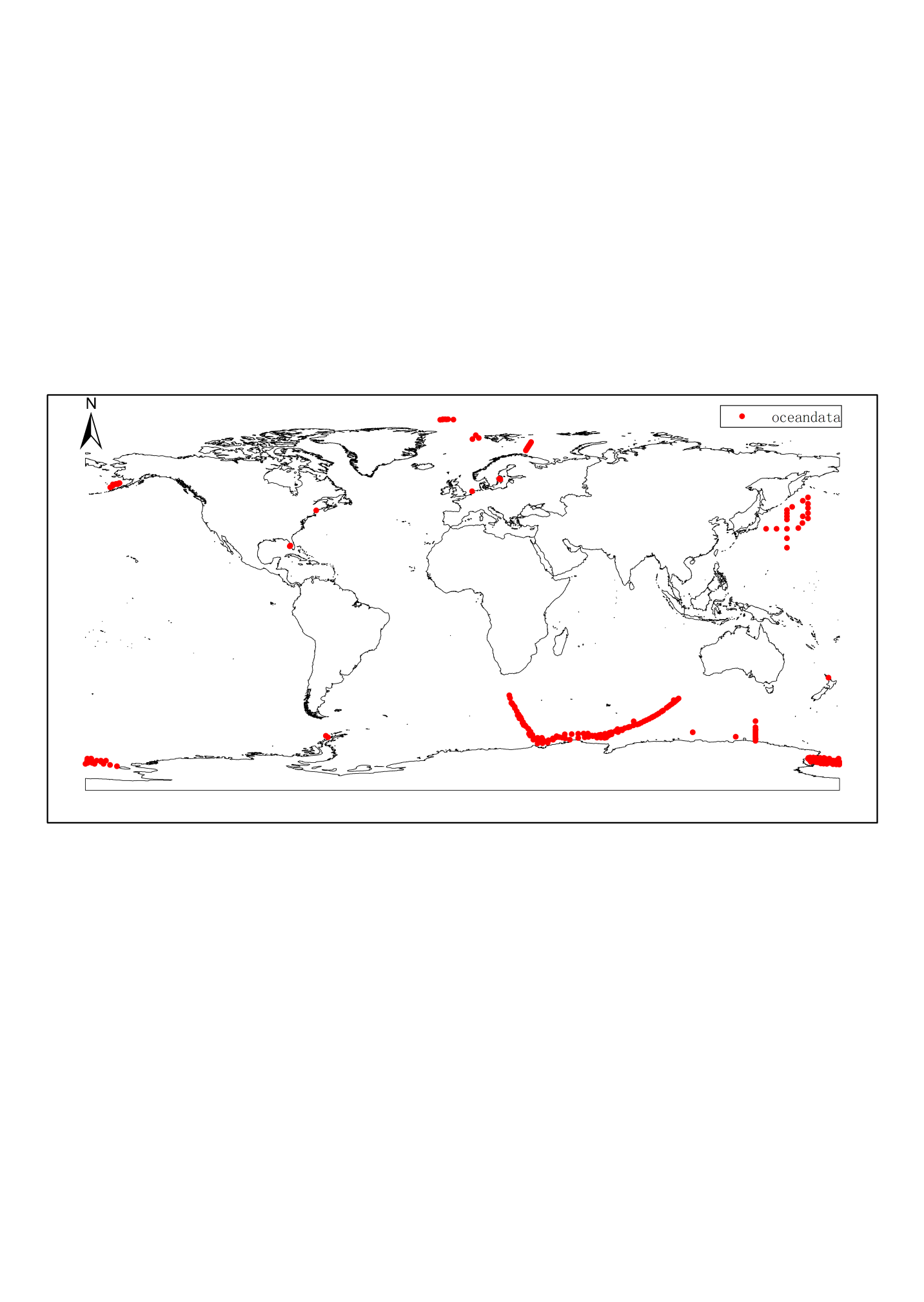


**Supplementary Figure 3. The distribution of sampling sites for the 426 data sets of surface oceans (including simultaneously detected DMS and Chl-a) from the global database of sea-surface DMS [37]. (**<https://saga.pmel.noaa.gov/dms/dms_data_out.php>**).**

**
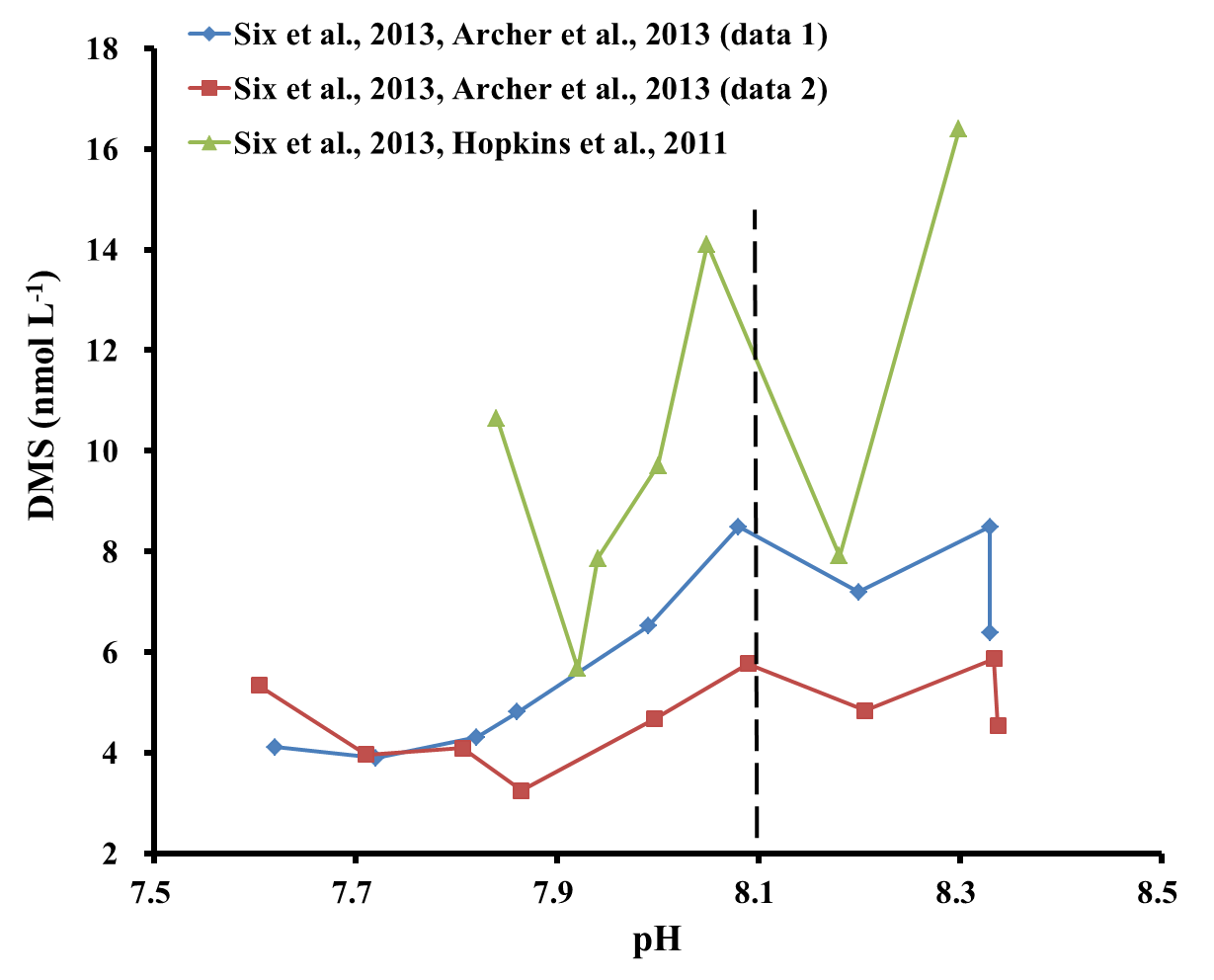
**

**Supplementary Figure 4. The relationships between DMS and pH in oceans from Six et al., 2013, Archer et al., 2013 and Hopkins et al., 2011, showing peak DMS concentrations at a pH of about 8.10 [8, 41, 59].**


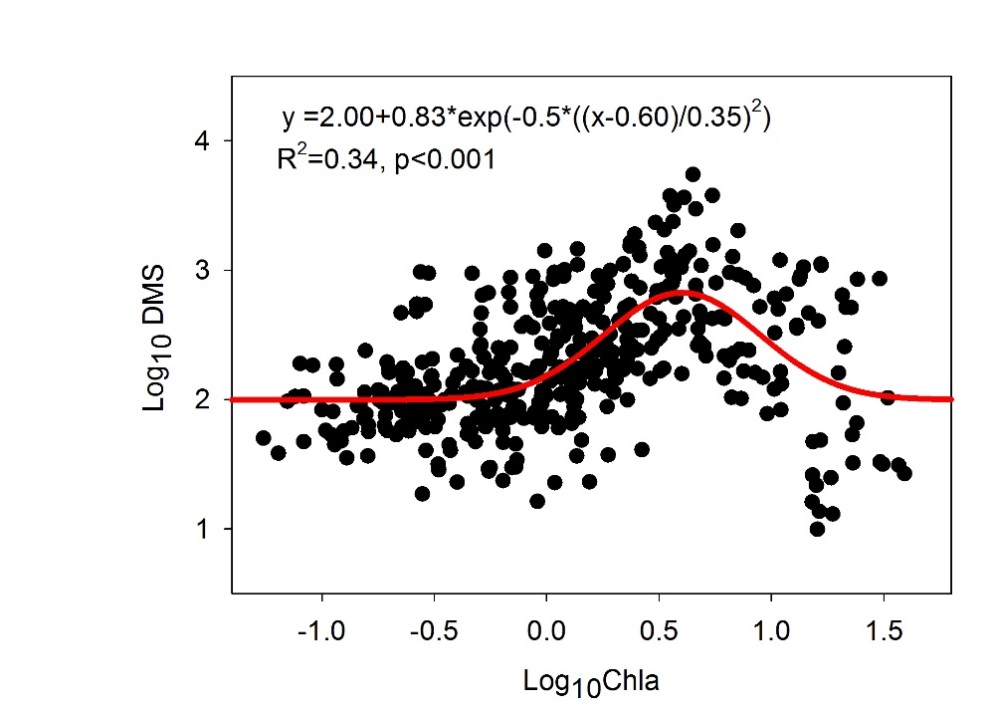


**Supplementary Figure 5. Empirical relationship between log-transformed dimethylsulfide (DMS ng L^-1^) concentrations and chlorophyll a (Chl-a mg m^-3^) in sea-surface water.**

**
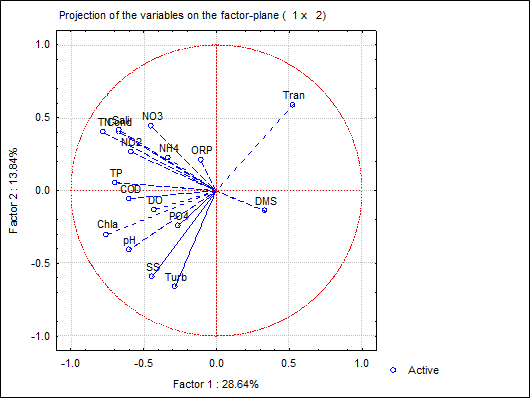
**

**Supplementary Figure 6. The relationships between DMS and environmental factors in 100 lakes through PCCA analysis. The result shows that DMS stands alone with others, Chla, pH and DO can be grouped together, and that various forms of nitrogen can be classified into one group. From PCCA analysis, the relationship between DO and DMS is likely to be similar with that between pH and DMS, likely because the changes of both DO and pH are mainly driven by algal photosynthesis. However, previous study showed that DO could not affect DMS directly [36], unless under the action of a catalyst. Therefore, environmental factors, such as DO, which may show spurious correlation with DMS, were not considered for further analysis.** Abbreviations are as follows: transparency - Tran, dissolved oxygen - DO, conductivity - Cond, salinity - Sali, turbidity - Turb, oxidation reduction potential - ORP, suspended solids - SS, total nitrogen - TN, nitrate - NO3, nitrite - NO2, ammonia - NH4, total phosphorus - TP, phosphate - PO4 and permanganate - COD.


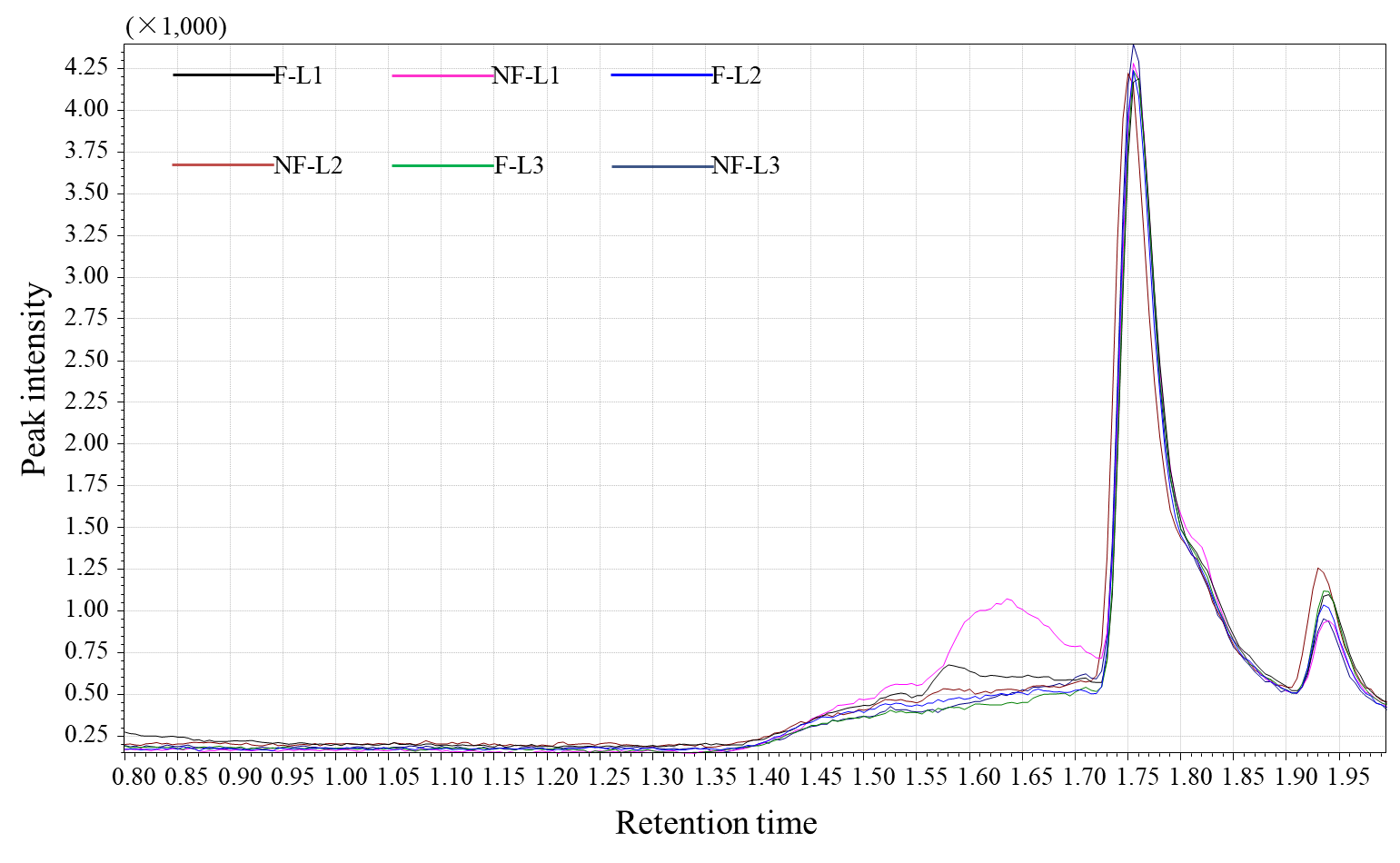


**Supplementary Figure 7. A comparison of GC-MS chromatograms of DMS between filtered lake waters (F-L1, F-L2, F-L3) and unfiltered lake waters (NF-L1, NF-L2, NF-L3).** Both the GC-MS chromatographs and RSD data (Supplementary Table 4) show that there is negligible difference in DMS concentrations between the two treatments.


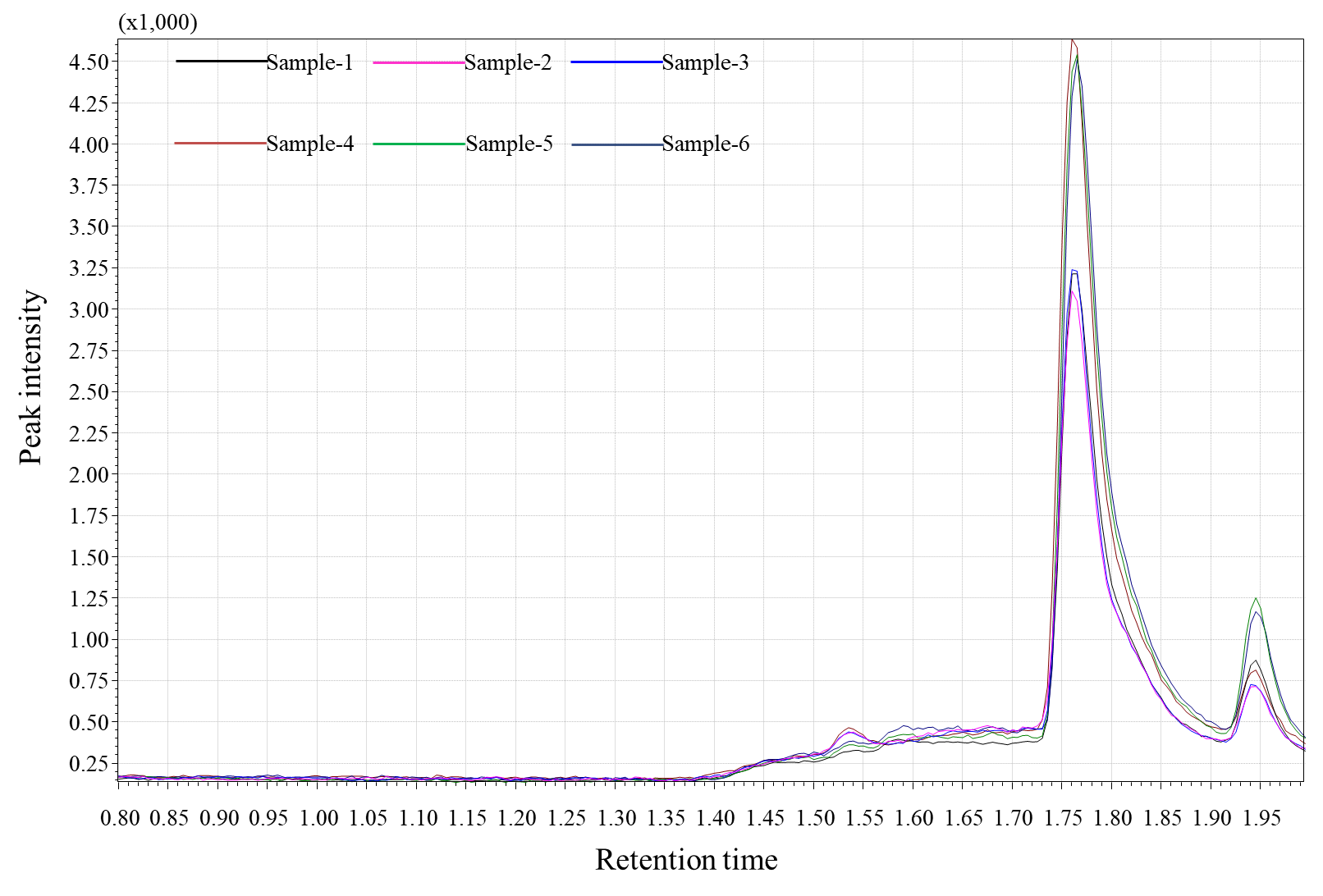


**Supplementary Figure 8.** **A comparison of GC-MS chromatograms of DMS between algal culture mediums (Sample-1 to Sample-3) and algal culture mediums with 10 M NaOH (Sample-4 to Sample-6).** The results showed that there is a significant difference between the two treatments.

**Supplementary Tables**

**Supplementary Table 1. Detailed information of 246 sites from the 100 lakes in the eastern plain of China.** Units of Chl-a and DMS are mg m^-3^ and ng L^-1^, respectively.

| **No.** | **Lat** | **Lon** | **pH** | **Chl-a** | **DMS** | **No.** | **Lat** | **Lon** | **pH** | **Chl-a** | **DMS** | **No.** | **Lat** | **Lon** | **pH** | **Chl-a** | **DMS** |
| --- | --- | --- | --- | --- | --- | --- | --- | --- | --- | --- | --- | --- | --- | --- | --- | --- | --- |
| 1 | 111.7358 | 29.0983 | 7.83 | 5.20 | 198.95 | 24 | 112.6147 | 31.1486 | 8.74 | 6.45 | 189.00 | 47 | 113.5508 | 29.7214 | 8.82 | 2.25 | 246.30 |
| 2 | 111.7420 | 29.0611 | 8.64 | 11.13 | 116.84 | 25 | 112.6283 | 31.1333 | 8.88 | 19.30 | 172.34 | 48 | 113.5558 | 29.7406 | 8.74 | 7.15 | 157.77 |
| 3 | 111.7603 | 29.0697 | 8.13 | 2.90 | 155.68 | 26 | 112.6354 | 29.3744 | 7.89 | 2.80 | 434.46 | 49 | 113.6494 | 30.8358 | 8.14 | 3.00 | 485.32 |
| 4 | 111.8814 | 29.7206 | 7.80 | 7.07 | 249.61 | 27 | 112.6440 | 29.3473 | 8.52 | 12.70 | 1044.91 | 50 | 113.6633 | 30.8142 | 8.52 | 21.47 | 331.51 |
| 5 | 111.9267 | 29.4353 | 8.88 | 20.20 | 259.15 | 28 | 112.6569 | 29.3697 | 8.99 | 11.00 | 401.15 | 51 | 113.6689 | 30.8556 | 8.25 | 8.63 | 181.20 |
| 6 | 111.9500 | 29.4181 | 7.70 | 4.45 | 178.82 | 29 | 112.9480 | 29.0708 | 8.96 | 25.18 | 161.44 | 52 | 113.8961 | 30.5050 | 8.34 | 12.03 | 350.82 |
| 7 | 111.9589 | 29.4036 | 8.37 | 6.77 | 175.71 | 30 | 113.0158 | 29.2487 | 8.94 | 4.20 | 96.81 | 53 | 113.9047 | 30.5086 | 8.60 | 2.30 | 500.00 |
| 8 | 111.9656 | 29.8367 | 8.49 | 3.20 | 286.66 | 31 | 113.0346 | 29.3044 | 9.10 | 4.90 | 116.22 | 54 | 114.0453 | 30.5502 | 8.48 | 6.77 | 650.23 |
| 9 | 111.9662 | 29.8189 | 9.30 | 8.33 | 149.49 | 32 | 113.0995 | 29.3378 | 8.94 | 23.40 | 124.21 | 55 | 114.0514 | 29.9364 | 8.30 | 10.00 | 464.95 |
| 10 | 112.0358 | 29.4308 | 9.50 | 22.75 | 18.54 | 33 | 113.1220 | 29.3347 | 8.60 | 16.55 | 105.40 | 56 | 114.0528 | 30.5444 | 8.82 | 2.35 | 769.82 |
| 11 | 112.0372 | 29.4183 | 9.59 | 29.95 | 14.03 | 34 | 113.1778 | 29.4433 | 8.13 | 3.93 | 156.83 | 57 | 114.0575 | 29.9589 | 8.72 | 12.63 | 290.83 |
| 12 | 112.0787 | 30.0572 | 9.57 | 58.00 | 7.91 | 35 | 113.1922 | 29.4408 | 8.23 | 16.30 | 203.34 | 58 | 114.0589 | 30.5383 | 8.00 | 8.30 | 880.89 |
| 13 | 112.1947 | 28.9239 | 9.11 | 14.00 | 148.44 | 36 | 113.2659 | 29.8913 | 7.97 | 4.53 | 108.85 | 59 | 114.0714 | 30.8431 | 8.94 | 22.05 | 174.29 |
| 14 | 112.2708 | 28.9264 | 7.79 | 2.50 | 511.53 | 37 | 113.2781 | 29.8583 | 8.60 | 24.07 | 222.18 | 60 | 114.0781 | 30.8608 | 9.18 | 14.70 | 130.27 |
| 15 | 112.3970 | 28.8293 | 7.99 | 1.20 | 59.50 | 38 | 113.3375 | 29.8361 | 8.95 | 22.38 | 79.96 | 61 | 114.0789 | 30.8786 | 9.07 | 12.97 | 196.46 |
| 16 | 112.4280 | 28.8045 | 8.16 | 7.30 | 260.03 | 39 | 113.3750 | 29.8489 | 8.14 | 3.30 | 761.39 | 62 | 114.0989 | 29.9778 | 8.12 | 1.37 | 277.01 |
| 17 | 112.4431 | 30.4542 | 8.09 | 7.00 | 693.59 | 40 | 113.3856 | 29.8272 | 7.78 | 5.00 | 843.65 | 63 | 114.1108 | 29.9589 | 8.00 | 8.23 | 440.60 |
| 18 | 112.4560 | 28.7932 | 8.54 | 4.80 | 259.76 | 41 | 113.3894 | 29.8758 | 8.31 | 2.30 | 202.94 | 64 | 114.1897 | 30.2111 | 8.12 | 2.15 | 284.17 |
| 19 | 112.4770 | 28.8643 | 7.90 | 3.20 | 107.60 | 42 | 113.4064 | 29.8986 | 8.07 | 3.70 | 260.90 | 65 | 114.1947 | 30.2028 | 8.07 | 4.60 | 921.71 |
| 20 | 112.4920 | 28.7944 | 8.11 | 3.45 | 236.35 | 43 | 113.4286 | 29.9211 | 7.72 | 3.27 | 211.00 | 66 | 114.2007 | 29.9401 | 8.13 | 6.75 | 296.71 |
| 21 | 112.5001 | 29.6435 | 8.29 | 11.10 | 233.13 | 44 | 113.5295 | 29.7045 | 9.03 | 10.67 | 89.84 | 67 | 114.2011 | 30.0506 | 8.59 | 10.15 | 290.16 |
| 22 | 112.5290 | 28.8120 | 8.53 | 8.65 | 286.32 | 45 | 113.5370 | 29.6853 | 8.15 | 8.20 | 149.65 | 68 | 114.2097 | 30.2247 | 8.17 | 3.12 | 823.95 |
| 23 | 112.5400 | 28.8395 | 9.14 | 8.97 | 253.08 | 46 | 113.5461 | 29.6700 | 8.57 | 10.03 | 232.39 | 69 | 114.2116 | 30.1312 | 7.98 | 8.00 | 59.94 |
| 70 | 114.2492 | 30.0026 | 7.52 | 2.50 | 71.74 | 95 | 114.7344 | 30.2300 | 8.47 | 9.83 | 58.68 | 120 | 115.8092 | 29.9922 | 8.76 | 18.15 | 29.39 |
| 71 | 114.2750 | 30.0061 | 8.56 | 11.37 | 323.64 | 96 | 115.0425 | 30.2939 | 7.45 | 5.45 | 77.13 | 121 | 115.8175 | 29.9700 | 9.00 | 18.67 | 59.43 |
| 72 | 114.2764 | 30.0061 | 8.16 | 2.35 | 253.82 | 97 | 115.0433 | 30.3058 | 8.80 | 10.80 | 84.09 | 122 | 115.8708 | 29.6839 | 8.44 | 7.40 | 1280.48 |
| 73 | 114.3398 | 30.4265 | 8.78 | 16.50 | 230.33 | 98 | 115.0452 | 30.2004 | 8.88 | 20.57 | 42.11 | 123 | 115.9789 | 38.9047 | 9.18 | 17.03 | 60.71 |
| 74 | 114.3583 | 30.5506 | 8.16 | 5.13 | 264.88 | 99 | 115.0599 | 30.2174 | 9.10 | 17.87 | 57.11 | 124 | 115.9858 | 38.9083 | 8.71 | 30.40 | 51.14 |
| 75 | 114.3706 | 30.4358 | 8.30 | 7.57 | 416.77 | 100 | 115.0619 | 30.1074 | 7.90 | 8.03 | 209.77 | 125 | 115.9992 | 38.9417 | 7.56 | 1.78 | 46.25 |
| 76 | 114.3781 | 30.5497 | 7.83 | 6.30 | 297.81 | 101 | 115.0861 | 30.1011 | 9.51 | 12.00 | 83.26 | 126 | 116.0019 | 38.9331 | 8.64 | 10.60 | 76.28 |
| 77 | 114.3814 | 30.4047 | 8.48 | 3.63 | 186.25 | 102 | 115.1147 | 30.0936 | 8.21 | 6.03 | 199.00 | 127 | 116.0461 | 29.2383 | 7.69 | 2.00 | 57.30 |
| 78 | 114.3878 | 30.4225 | 8.21 | 6.00 | 108.00 | 103 | 115.1389 | 30.0906 | 8.92 | 13.73 | 107.80 | 128 | 116.0519 | 28.6847 | 9.44 | 23.85 | 24.54 |
| 79 | 114.3997 | 30.5772 | 8.59 | 12.00 | 352.55 | 104 | 115.2450 | 30.0568 | 9.60 | 15.17 | 92.75 | 129 | 116.0590 | 29.4475 | 8.04 | 2.60 | 102.62 |
| 80 | 114.4703 | 30.5742 | 8.22 | 6.10 | 251.90 | 105 | 115.2457 | 30.0586 | 7.42 | 2.00 | 26.92 | 130 | 116.0930 | 29.4706 | 8.33 | 8.60 | 116.95 |
| 81 | 114.4736 | 30.5711 | 8.39 | 16.00 | 401.94 | 106 | 115.3844 | 30.0875 | 8.82 | 14.00 | 114.28 | 131 | 116.1010 | 29.2493 | 8.70 | 19.43 | 69.96 |
| 82 | 114.4847 | 30.8017 | 7.92 | 10.25 | 460.53 | 107 | 115.3853 | 30.1436 | 7.80 | 2.00 | 96.08 | 132 | 116.1138 | 29.9231 | 9.17 | 15.03 | 192.64 |
| 83 | 114.4869 | 30.2483 | 7.45 | 1.20 | 31.15 | 108 | 115.3889 | 29.8286 | 8.31 | 7.45 | 199.69 | 133 | 116.1365 | 29.9539 | 8.54 | 10.60 | 164.65 |
| 84 | 114.4906 | 30.7867 | 8.66 | 10.00 | 654.83 | 109 | 115.3964 | 30.1339 | 9.20 | 18.90 | 127.38 | 134 | 116.1513 | 29.3694 | 8.28 | 4.05 | 191.59 |
| 85 | 114.4993 | 30.8034 | 8.71 | 7.60 | 282.83 | 110 | 115.4006 | 29.8378 | 8.39 | 18.43 | 96.55 | 135 | 116.1544 | 35.9586 | 9.36 | 6.33 | 103.72 |
| 86 | 114.5492 | 30.4161 | 8.38 | 7.65 | 313.11 | 111 | 115.5825 | 29.9211 | 9.27 | 12.80 | 115.08 | 136 | 116.1610 | 29.3656 | 8.81 | 10.80 | 287.09 |
| 87 | 114.5792 | 30.2769 | 8.05 | 1.40 | 48.90 | 112 | 115.5848 | 29.9072 | 9.25 | 9.27 | 108.94 | 137 | 116.1736 | 29.3600 | 8.09 | 1.85 | 257.88 |
| 88 | 114.5839 | 30.2022 | 8.67 | 13.53 | 47.12 | 113 | 115.5969 | 29.9003 | 8.45 | 23.87 | 87.67 | 138 | 116.1834 | 29.9352 | 7.75 | 3.25 | 147.18 |
| 89 | 114.5842 | 30.2322 | 8.86 | 9.77 | 54.22 | 114 | 115.6225 | 37.6467 | 9.60 | 18.80 | 44.70 | 139 | 116.1878 | 36.0214 | 8.50 | 2.15 | 72.30 |
| 90 | 114.6881 | 30.6536 | 8.02 | 2.35 | 295.03 | 115 | 115.6383 | 37.6500 | 8.39 | 18.85 | 61.18 | 140 | 116.1894 | 35.9872 | 8.51 | 11.00 | 62.06 |
| 91 | 114.6999 | 30.6356 | 8.90 | 3.23 | 166.26 | 116 | 115.6929 | 29.7836 | 7.80 | 2.50 | 462.96 | 141 | 116.1897 | 29.9681 | 7.69 | 0.65 | 33.76 |
| 92 | 114.7028 | 30.2458 | 9.35 | 10.00 | 63.15 | 117 | 115.6989 | 29.7767 | 7.73 | 5.00 | 488.15 | 142 | 116.1900 | 29.7094 | 9.28 | 12.73 | 94.95 |
| 93 | 114.7164 | 30.6411 | 8.81 | 11.00 | 53.52 | 118 | 115.7098 | 29.7858 | 7.95 | 6.97 | 606.26 | 143 | 116.1994 | 36.0544 | 9.57 | 29.20 | 19.71 |
| 94 | 114.7250 | 30.2583 | 8.05 | 10.00 | 80.00 | 119 | 115.7967 | 29.9545 | 9.21 | 23.20 | 24.22 | 144 | 116.2236 | 35.9972 | 7.99 | 8.83 | 95.19 |
| 145 | 116.2350 | 32.3314 | 8.99 | 30.50 | 42.35 | 170 | 116.4673 | 30.1492 | 8.72 | 1.80 | 58.80 | 195 | 117.1153 | 30.7772 | 7.69 | 3.12 | 95.64 |
| 146 | 116.2417 | 35.9972 | 9.14 | 10.05 | 223.75 | 171 | 116.5928 | 32.5931 | 8.08 | 1.83 | 500.00 | 196 | 117.1219 | 30.7893 | 8.31 | 13.00 | 195.21 |
| 147 | 116.2848 | 29.1980 | 9.05 | 12.85 | 48.39 | 172 | 116.6006 | 32.6092 | 8.80 | 13.86 | 61.48 | 197 | 117.1383 | 32.7144 | 9.18 | 20.90 | 35.56 |
| 148 | 116.2997 | 28.5089 | 7.36 | 1.08 | 18.01 | 173 | 116.6086 | 35.2572 | 9.43 | 32.10 | 13.69 | 198 | 117.1561 | 30.6454 | 8.87 | 20.95 | 152.98 |
| 149 | 116.3040 | 28.5669 | 7.29 | 0.55 | 12.05 | 174 | 116.6156 | 32.6017 | 8.40 | 17.70 | 58.54 | 199 | 117.1673 | 30.6562 | 8.79 | 4.10 | 134.27 |
| 150 | 116.3067 | 29.0936 | 8.94 | 12.57 | 96.12 | 175 | 116.6498 | 29.1294 | 7.73 | 1.01 | 28.03 | 200 | 117.1695 | 30.6286 | 8.51 | 23.95 | 103.86 |
| 151 | 116.3123 | 28.6025 | 7.60 | 1.85 | 28.51 | 176 | 116.6528 | 35.2172 | 7.64 | 2.92 | 86.89 | 201 | 117.1827 | 30.6703 | 8.94 | 37.25 | 107.43 |
| 152 | 116.3214 | 30.0300 | 8.58 | 24.50 | 67.82 | 177 | 116.6652 | 29.1496 | 8.68 | 4.30 | 123.54 | 202 | 117.3342 | 31.6244 | 9.47 | 40.00 | 22.69 |
| 153 | 116.3280 | 28.5499 | 7.70 | 1.08 | 52.05 | 178 | 116.6689 | 35.1511 | 7.60 | 2.82 | 109.48 | 203 | 117.3353 | 31.6767 | 9.47 | 52.00 | 15.22 |
| 154 | 116.3300 | 28.5063 | 7.48 | 1.55 | 27.64 | 179 | 116.6831 | 32.3275 | 9.67 | 22.00 | 34.00 | 204 | 117.3889 | 31.6017 | 9.50 | 37.00 | 23.40 |
| 155 | 116.3381 | 30.0500 | 7.98 | 11.10 | 73.02 | 180 | 116.7616 | 30.2765 | 9.07 | 15.70 | 35.72 | 205 | 117.4161 | 31.6458 | 9.30 | 10.20 | 165.50 |
| 156 | 116.3410 | 30.0068 | 8.85 | 2.50 | 67.07 | 181 | 116.7875 | 35.0606 | 9.30 | 9.10 | 147.94 | 206 | 117.5133 | 31.4858 | 9.73 | 33.00 | 23.45 |
| 157 | 116.3541 | 30.2047 | 7.89 | 1.90 | 54.90 | 182 | 116.8014 | 35.0392 | 8.58 | 24.30 | 59.47 | 207 | 117.5603 | 31.5425 | 9.45 | 39.00 | 16.58 |
| 158 | 116.3548 | 28.5421 | 7.55 | 1.15 | 21.93 | 183 | 116.9061 | 32.3403 | 8.18 | 1.80 | 258.41 | 208 | 117.6536 | 33.1339 | 8.78 | 2.30 | 53.34 |
| 159 | 116.3573 | 29.0810 | 8.83 | 42.10 | 82.68 | 184 | 116.9069 | 32.3872 | 8.90 | 41.80 | 46.31 | 209 | 117.8314 | 32.9828 | 8.05 | 2.03 | 101.67 |
| 160 | 116.3642 | 30.0289 | 8.07 | 4.35 | 155.66 | 185 | 116.9069 | 34.9483 | 8.48 | 19.57 | 57.17 | 210 | 117.8394 | 33.1767 | 8.88 | 32.85 | 146.87 |
| 161 | 116.3653 | 28.6545 | 8.31 | 4.63 | 91.53 | 186 | 116.9119 | 32.3653 | 7.83 | 8.80 | 284.43 | 211 | 117.9119 | 33.2878 | 9.47 | 46.00 | 34.08 |
| 162 | 116.3728 | 28.6461 | 7.94 | 9.95 | 79.54 | 187 | 116.9806 | 34.8931 | 8.83 | 12.00 | 49.76 | 212 | 117.9514 | 33.2142 | 8.52 | 13.90 | 232.89 |
| 163 | 116.3747 | 28.5356 | 7.39 | 0.73 | 19.12 | 188 | 116.9806 | 34.8931 | 8.57 | 11.93 | 224.61 | 213 | 118.0011 | 32.8953 | 8.17 | 2.15 | 231.96 |
| 164 | 116.3828 | 28.6642 | 8.53 | 12.95 | 95.27 | 189 | 117.0036 | 34.8406 | 7.63 | 1.30 | 22.60 | 214 | 118.1117 | 33.0081 | 8.30 | 20.87 | 64.65 |
| 165 | 116.4029 | 29.0391 | 9.05 | 26.28 | 72.62 | 190 | 117.0953 | 30.8232 | 8.66 | 20.20 | 105.25 | 215 | 118.5447 | 33.2953 | 8.24 | 3.07 | 197.85 |
| 166 | 116.4215 | 30.0284 | 9.18 | 11.40 | 226.42 | 191 | 117.0959 | 30.8424 | 8.53 | 17.07 | 206.89 | 216 | 118.8647 | 31.4746 | 8.84 | 2.75 | 129.61 |
| 167 | 116.4320 | 30.0152 | 8.79 | 13.75 | 45.73 | 192 | 117.0971 | 30.8325 | 8.27 | 4.00 | 105.02 | 217 | 118.8901 | 31.4930 | 8.04 | 2.80 | 180.27 |
| 168 | 116.4457 | 29.9988 | 7.57 | 1.00 | 70.65 | 193 | 117.1005 | 30.8107 | 9.40 | 11.63 | 164.88 | 218 | 118.8902 | 31.3096 | 8.60 | 18.00 | 43.62 |
| 169 | 116.4653 | 29.9885 | 8.95 | 45.00 | 59.53 | 194 | 117.1071 | 30.7980 | 8.54 | 17.90 | 103.33 | 219 | 118.8940 | 31.5041 | 7.78 | 1.60 | 40.66 |
| 220 | 118.8960 | 31.4572 | 8.98 | 14.35 | 53.95 | 229 | 119.5938 | 31.6499 | 8.85 | 24.15 | 163.82 | 238 | 120.7460 | 31.6006 | 7.48 | 1.80 | 67.84 |
| 221 | 118.9146 | 31.2914 | 7.60 | 8.50 | 48.58 | 230 | 119.5944 | 31.6182 | 9.03 | 1.90 | 46.45 | 239 | 120.7460 | 31.6006 | 8.54 | 3.45 | 103.81 |
| 222 | 118.9405 | 31.5128 | 7.70 | 5.45 | 105.51 | 231 | 119.7606 | 31.3808 | 7.74 | 1.50 | 65.03 | 240 | 120.7494 | 31.5799 | 8.49 | 26.53 | 50.48 |
| 223 | 118.9430 | 31.4633 | 7.53 | 5.73 | 94.46 | 232 | 119.8028 | 31.3698 | 8.51 | 33.95 | 57.26 | 241 | 120.7968 | 31.4720 | 8.98 | 2.70 | 41.57 |
| 224 | 119.1200 | 33.2850 | 8.66 | 14.00 | 53.78 | 233 | 119.8057 | 31.6388 | 8.88 | 47.60 | 22.96 | 242 | 120.8108 | 31.4084 | 8.92 | 11.20 | 47.66 |
| 225 | 119.2189 | 32.7958 | 9.84 | 49.00 | 7.79 | 234 | 119.8183 | 33.1558 | 8.50 | 2.00 | 53.08 | 243 | 120.8122 | 31.1965 | 9.13 | 39.85 | 108.36 |
| 226 | 119.5311 | 31.6275 | 9.01 | 2.30 | 74.70 | 235 | 119.8264 | 33.1522 | 7.91 | 2.97 | 113.03 | 244 | 120.8249 | 31.2146 | 8.93 | 27.00 | 107.51 |
| 227 | 119.5475 | 31.6125 | 8.22 | 13.80 | 169.82 | 236 | 119.8543 | 31.6679 | 7.50 | 0.95 | 131.95 | 245 | 120.8532 | 31.2180 | 7.81 | 6.10 | 69.13 |
| 228 | 119.5593 | 31.6261 | 8.88 | 6.83 | 84.66 | 237 | 120.7393 | 31.4523 | 8.94 | 10.70 | 144.80 | 246 | 120.8977 | 31.0684 | 8.62 | 9.78 | 74.40 |

**Supplementary Table 2. Detailed information of 426 datasets from the global database of simultaneously detected sea-surface DMS and Chl-a [37].** (https://saga.pmel.noaa.gov/dms/dms_data_out.php).

| **Contributor** | **Platform** | **Region** | **Reference(s)** |
| --- | --- | --- | --- |
| Andreae | unknown ship | Bering Sea | Barnard et al., 1984 [23] |
| Andreae | Bellows | Charlotte Harbour | Froelich et al., 1985 [21] |
| Kettle_D | station B1 | Baltic Sea | Leck et al., 1990 [20] |
| Kettle_D | helicopter | Baltic Sea | Leck et al., 1990 [20] |
| Kettle_D | Hakuho-Maru | Pacific | Watanabe et al., 1995 [22] |
| Keller | unknown ship | Gulf of Maine | unpublished |
| Kettle_D | Weatherbird | Sargasso Sea | Siegel and Michaels, 1996 [60] |
| Matrai | Jan Mayen | Barents Sea | Matrai and Vernet, 1997 [56] |
| Kettle_D | from shore | New Zealand | Lee and de Mora, 1996 [58] |
| Lee, DiTullio | Nathaniel B. Palmer | Ross Sea | unpublished |
| Lee, DiTullio | Nathaniel B. Palmer | Ross Sea | unpublished |
| Kasamatsu | RT/V Umitaka Maru | Southern Ocean (Indian sector) | unpublished |
| Kasamatsu | RT/V Umitaka Maru | Southern Ocean (Indian sector) | unpublished |
| Kasamatsu | R/V Mirai (JAMSTEC) | Western North Pacific | unpublished |
| Kasamatsu | RT/V Tangaroa | Southern Ocean | Kasamatsu et al., 2004; 2005 [53-54] |
| Kasamatsu | RT/V Umitaka Maru | Southern Ocean (Indian sector) | unpublished |
| Kasamatsu | RT/V Umitaka Maru | Southern Ocean | Preunkert et al., 2007; Kasamatsu et al., 2004; 2005 [52-54] |
| Kasamatsu | RT/V Tangaroa | Southern Ocean | Preunkert et al., 2007; Kasamatsu et al., 2004; 2005 [52-54] |
| Matrai, Dacey | Zodiac | Western Antarctic Peninsula | unpublished |
| Matrai | I/B Oden | Arctic Ocean | Orellana et. al., 2011; Tjernstrom et al 2012 [51, 55] |

**Supplementary Table 3. Comparison of global maps of sea surface DMS.**

| Annual mean DMS  (nmol L^-1^) | Methods | Reference |
| --- | --- | --- |
| 2.13 | Compilation of DMS data Extrapolations Interpolations | Kettle et al., 1999 [61] |
| 2.01 | Updated compilation of DMS data Extrapolations Interpolations | Kettle and Andreae, 2000 [10] |
| 2.55 | Parameterizations: Chl-a, nitrates, light | Anderson et al., 2001 [43] |
| 1.70 | Process model parameterizations: Chl-a, Fp-ratio | Aumont et al., 2002 [44] |
| 2.28 | Parameterizations: Chl-a | Simo and Dachs, 2002 [45] |
| 1.51 | Process model | Chu et al., 2003[46] |
| 1.60 | Parameterizations: Chl-a, Fp-ratio | Belviso et al., 2004 [19] |
| **1.92** | **Parameterizations: Chl-a** | **This study** |

**Supplementary Table 4.** **The peak areas were calculated by automatic peak operation under the same condition by GCMS solution, and the average filtration loss rate and recovery were calculated by average peak areas.** RSD of the filtered group (F-L1, F-L2, and F-L3) and unfiltered group (NF-L1, NF-L2, and NF-L3) are 2.0% and 2.2%, respectively, and RSD of the six samples is 1.9%.

| Sample | Peak area | Sample | Peak area |
| --- | --- | --- | --- |
| F-L1 | 5436 | NF-L1 | 5261 |
| F-L2 | 5242 | NF-L2 | 5173 |
| F-L3 | 5266 | NF-L3 | 5402 |
| Average peak area | 5314.7 | Average peak area | 5278.7 |
| RSD | 2.0% | RSD | 2.2% |
| RSD of all samples | 1.9% | | |
| Average filtration loss rate | -0.7% | | |
| Recovery | 100.7% | | |

**Supplementary Table 5.** **DMS, total DMSP, algal density and Chl-a concentrations in the algal culture mediums.** Sample-1 to Sample-3 were 3 mL of the algal culture mediums diluted with 3 mL ultrapure water, respectively. Sample-4 to Sample-6 were 3 mL of the algal culture mediums diluted with 0.25 mL 10 M NaOH and 2.75 mL ultrapure water. Total DMSP was calculated by the difference of the average DMS concentrations in the two treatments. Algal density and Chl-a values were measured in the algal culture medium after double dilution.

| Sample | DMS  (ng L^-1^) | DMS  Mean ± SD  (ng L^-1^) | Total DMSP  (nM L^-1^) | Algal density  (cells L^-1^) | Chl-a  (μg L^-1^) |
| --- | --- | --- | --- | --- | --- |
| Sample-1 | 56.08 | 55.35±1.88 | 0.48 | 9×10^9^ | 116.6 |
| Sample-2 | 53.22 |  |  |  |  |
| Sample-3 | 56.76 |  |  |  |  |
| Sample-4 | 84.22 | 85.17±3.70 |  |  |  |
| Sample-5 | 82.04 |  |  |  |  |
| Sample-6 | 89.25 |  |  |  |  |

**Supplementary Table 6.** **DMSP concentrations in filtrate and filters.** Filtrate-1 to Filtrate-3 were 2 mL filtrate diluted with 2 mL 10 M NaOH and 40 mL ultrapure water, and Filter-1 to Filter-3 were 2 mL algae on filters diluted with 2 mL 10 M NaOH and 42 mL ultrapure water. (a) is the value of dissolved DMSP that calculated by DMS in filtrates and culture mediums, (b) is the value of intracellular DMSP.

| Samples | DMS  (ng L^-1^) | DMS  Mean ± SD  (ng L^-1^) | DMSP  (nM L^-1^) | Algal number  (cells) |
| --- | --- | --- | --- | --- |
| Filtrate-1 | 8.29 | 7.48 ± 0.70 | 0.04±0.01  (a) | - |
| Filtrate-2 | 7.12 |  |  |  |
| Filtrate-3 | 7.03 |  |  |  |
| Filter-1 | 5.18 | 5.28 ± 0.19 | 0.085±0.003  (b) | 4.5×10^6^ |
| Filter-2 | 5.50 |  |  |  |
| Filter-3 | 5.15 |  |  |  |

**Supplementary References**

51. Orellana MV, Matrai PA, Leck C *et al.* Rauschenberg, A. M. Lee, E. Coz, Marine microgels as a source of cloud condensation nuclei in the high Arctic. *Proc Natl Acad Sci USA* 2011; **108**: 13612-7.

52. Preunkert S, Legrand M, Jourdain B *et al.* Interannual variability of dimethylsulfide in air and seawater and its atmospheric oxidation by-products (methanesulfonate and sulfate) at Dumont d'Urville, coastal Antarctica (1999-2003). *J Geophys Res* 2007; **112**: D06306.

53. Kasamatsu N, Kawaguchi S, Watanabe S *et al.* Possible impacts of zooplankton grazing on dimethylsulfide production in the Antarctic Ocean. *Can J Fish Aquat Sci* 2004; **61**: 736-43.

54. Kasamatsu N, Odate T, Fukuchi M. Dimethylsulfide and Dimethylsulfoniopropionate Production in the Antarctic Pelagic Food Web. *Ocean Polar Res* 2005; **27**: 197-203.

55. Tjernström M, Leck C, Birch CE *et al.* The Arctic Summer Cloud Ocean Study (ASCOS): overview and experimental design. *Atmos Chem Phys* 2014; **14**: 2823-69.

56. Matrai PA, Vernet M. Dynamics of the vernal bloom in the marginal ice-zone of the Barents Sea. DMS and DMSP budgets. *J Geophys Res* 1997; **102**: 22965-71.

57. Kwint RLJ, Kramer KJM. Annual cycle of the production and fate of DMS and DMSP in the marine coastal system. *Mar Ecol Prog Ser* 1996; **134**: 217-24.

58. Lee PA, de Mora SJ. DMSP, DMS and DMSO concentrations and temporal trends in marine surface waters at Leigh, New Zealand, in Biological and Environmental Chemistry of DMSP and Related Sulfonium Compounds. In: Kiene RP, Visscher PT, Keller MD *et al.* (ed.). Plenum, New York, 1996, 391-404.

59. Hopkins F, Nightingale P, Liss P. Effect of ocean acidification on the marine source of atmospherically active trace gases, in Ocean Acidification. In: Gattuso JP, Hansson L (ed.). , Oxford Univ. Press, 2011, 210-229.

60. Siegel DA, Michaels AF. Quantification of non-algal light attenuation in the Sargasso Sea: Implications for biogeochemistry and remote sensing. *Deep Sea Res* 1996; **44**: 321-45.

61. Kettle AJ, Andreae MO, Amouroux D *et al.* A global database of sea surface dimethylsulfide (DMS) measurements and a procedure to predict sea surface DMS as a function of latitude, longitude, and month. *Glob Biogeochem Cycle* 1999; **13**: 399-444.
